# Performance of Computer-Aided Diagnosis in Ultrasonography for Detection of Breast Lesions Less and More Than 2 cm: Prospective Comparative Study

**DOI:** 10.2196/16334

**Published:** 2020-03-02

**Authors:** Liang Yongping, Ping Zhou, Zhang Juan, Zhao Yongfeng, Wengang Liu, Yifan Shi

**Affiliations:** 1 The Third Xiangya Hospital Central South University Changsha, Hunan China

**Keywords:** ultrasonography, breast neoplasm, breast imaging reporting and data system (BI-RADS), breast neoplasms diagnosis, cancer screening, computer diagnostic aid

## Abstract

**Background:**

Computer-aided diagnosis (CAD) is used as an aid tool by radiologists on breast lesion diagnosis in ultrasonography. Previous studies demonstrated that CAD can improve the diagnosis performance of radiologists. However, the optimal use of CAD on breast lesions according to size (below or above 2 cm) has not been assessed.

**Objective:**

The aim of this study was to compare the performance of different radiologists using CAD to detect breast tumors less and more than 2 cm in size.

**Methods:**

We prospectively enrolled 261 consecutive patients (mean age 43 years; age range 17-70 years), including 398 lesions (148 lesions>2 cm, 79 malignant and 69 benign; 250 lesions≤2 cm, 71 malignant and 179 benign) with breast mass as the prominent symptom. One novice radiologist with 1 year of ultrasonography experience and one experienced radiologist with 5 years of ultrasonography experience were each assigned to read the ultrasonography images without CAD, and then again at a second reading while applying the CAD S-Detect. We then compared the diagnostic performance of the readers in the two readings (without and combined with CAD) with breast imaging. The McNemar test for paired data was used for statistical analysis.

**Results:**

For the novice reader, the area under the receiver operating characteristic curve (AUC) improved from 0.74 (95% CI 0.67-0.82) from the without-CAD mode to 0.88 (95% CI 0.83-0.93; *P*<.001) at the combined-CAD mode in lesions≤2 cm. For the experienced reader, the AUC improved from 0.84 (95% CI 0.77-0.90) to 0.90 (95% CI 0.86-0.94; *P*=.002). In lesions>2 cm, the AUC moderately decreased from 0.81 to 0.80 (novice reader) and from 0.90 to 0.82 (experienced reader). The sensitivity of the novice and experienced reader in lesions≤2 cm improved from 61.97% and 73.23% at the without-CAD mode to 90.14% and 97.18% (both *P*<.001) at the combined-CAD mode, respectively.

**Conclusions:**

S-Detect is a feasible diagnostic tool that can improve the sensitivity for both novice and experienced readers, while also improving the negative predictive value and AUC for lesions≤2 cm, demonstrating important application value in the clinical diagnosis of breast cancer.

**Trial Registration:**

Chinese Clinical Trial Registry ChiCTR1800019649; http://www.chictr.org.cn/showprojen.aspx?proj=33094

## Introduction

Breast cancer is one of the most common cancers in women, and the second leading cause of cancer-related mortality worldwide [1,2]. Early diagnosis of breast cancer can increase the treatment options and survival rate of patients [3], in which breast ultrasound plays an important role in detecting breast cancer. Operator experience-dependence remains the main limitation in ultrasound-based diagnosis [4,5]. S-detect is a recently developed computer-assisted diagnosis (CAD) system for breast cancer, which is based on the Breast Imaging Reporting and Data System (BI-RADS) lexicon and classification [6]. Many studies have reported that S-detect has potential to become a novel diagnostic tool for radiologists [7-10]. However, no study has evaluated the diagnosis performance of CAD in breast lesions with respect to size (less and more than 2 cm). Therefore, the purpose of this study was to compare the performance of detecting breast cancer using CAD between radiologists with different levels of experience for lesions greater and less than 2 cm in size.

## Methods

### Patient Selection

We prospectively enrolled 261 patients who presented with a total of 398 lesions from November 2018 to May 2019. All patients underwent ultrasound before surgery. The mean age of the examined patients was 43.11 (SD 12.55) years (range 17-70 years). The diameter of lesions ranged from 0.26 to 9.50 cm, with a mean diameter of 1.92 (SD 1.26) cm. All 398 lesions were examined after surgery to confirm their pathological type. This prospective study was approved by the Institutional Review Board of Third Xiangya Hospital. Informed consent was obtained from all patients.

The inclusion criteria were follows: patients aged 17-70 years with breast tumor requiring surgery. The exclusion criteria were a history of neoadjuvant chemotherapy or endocrine therapy before surgery, lesions punctured by core-needle biopsy or Mammotome System, breast equipped with a prosthesis, lesions unclear as displayed by ultrasound, and patients unwilling to take part in the study.

### Ultrasound Image Acquisition

All images were obtained with an RS80A ultrasound system (Samsung Medison Co Ltd, Seoul, Korea) with a 5-13–MHz bandwidth linear transducer. All ultrasound examinations were performed by an independent radiologist with 3 years of experience. Typical images of the tumor in longitudinal and transverse planes were stored in the ultrasound system.

### Computer-Assisted Diagnostic System

Our CAD system (S-Detect) extracts features using an integration of artificial neural network classifiers internally installed in the ultrasound equipment (RS80A). The sensitivity of the instrument can be adjusted, with greater sensitivity yielding a higher potential rate of false-positive findings. We chose the default setting. To test the reproducibility of CAD marks with the same image, we randomly selected 20 of 398 (5.0%) examinations, which were sent through the CAD system three times, and the results showed that the markings were consistent in all images.

In S-Detect, the cursor on the center of the lesion was identified, and a region of interest was drawn along the border of the mass automatically by the ultrasound system. The ultrasound features of the lesion were analyzed according to the BI-RADS lexicon, and the final assessment classifications were automatically performed by the ultrasound system. If the borderline was considered inaccurate in any area of the tumor, it was manually edited to achieve the optimum fitness. In the S-Detect system, the final assessment classification was divided into “possibly benign” or “possibly malignant.”

### Diagnostic Criteria

According to the fifth version of BI-RADS, the radiologists classified the lesion from BI-RADS category 3 to BI-RADS category 5. BI-RADS category 4 was further subdivided into category 4A, 4B, and 4C. Category 3 is considered probably benign (<2% likelihood of malignancy) and categories 4A, 4B, 4C range from low to high suspicion (2-10%, 10-50%, 50-95% likelihood of malignancy, respectively). Category 5 indicates a high malignancy rate (>95% likelihood of malignancy). The malignant signs in breast ultrasound imaging included irregular shape, antiparallel orientation, noncircumscribed margin, microcalcification, acoustic halo, posterior shadowing, and abnormalities of the surrounding tissue. No definitive malignant sign is assigned to category 3; one, two, and three malignant signs are assigned to category 4A, 4B, and 4C, respectively; and more than four malignant signs is assigned to category 5. Accordingly, category 3 and 4A lesions were regarded as benign, and category 4B, 4C, and 5 lesions were regarded as malignant [11,12].

For assessments of the combination of ultrasound and the CAD system, we took longitudinal and transverse planes of the tumor for CAD. If one plane indicated “possibly malignant,” it was considered a positive outcome, and the BI-RADS category diagnosis was increased by one level (ie, 3 to 4A, 4A to 4B, 4B to 4C, 4C to 5). If both planes indicated “possibly benign,” it was considered a negative outcome, and the BI-RADS category diagnosis was decreased by one level (ie, 5 to 4C, 4C to 4B, 4B to 4A, 4A to 3) [13].

### Readers, Reading Modes, and Training

Two readers were involved in the study: a novice reader with 1 year of ultrasound experience and an experienced reader with 5 years of ultrasound experience. Both readers were trained on the reading procedures with 20 ultrasound images that were not part of the study set, 10 of which were read in without-CAD mode. The other 10 images were assessed in combined-CAD mode, in which the readers first read the ultrasound images without CAD and then combined the indications of CAD marks to make the final decision.

Both readers reviewed every examination at each reading mode independently and were blinded to any information about the patients, including age, manifestation of symptoms, and previous radiology reports. The readers were asked to read for at least 2 hours a day to simulate the typical process of batch reading in such examinations.

### Statistical Analysis

Statistical evaluation was performed using SPSS software (SPSS for Windows 19.0, SPSS Inc, Chicago, IL, USA). Taking the pathology results as the gold standard, we analyzed the diagnostic sensitivity, specificity, and area under the receiving operating characteristic curve values (AUCs) in without-CAD mode and combined-CAD mode [14]. The combined-CAD mode and without-CAD mode diagnostic parameters were compared using the McNemar test (sensitivity, specificity, positive predictive value [PPV], negative predictive value [NPV], accuracy) for match-paired data. We used the Hanley and McNeil method to analyze the differences between pairs of AUCs. For all statistical tests, *P*<.05 was considered to indicate statistical significance.

## Results

### Basic Characteristics of Lesions

Patient and lesion characteristics on the basis of lesion size are summarized in Table 1. Of the 398 breast lesions in the 261 patients included in this study, 250 (62.8%) were ≤2 cm and 148 (37.2%) were >2 cm. The mean sizes for all lesions, malignant lesions, and benign lesions at ultrasound were similar and close to 2 cm, with benign lesions being the smallest (1.73 cm) and malignant lesions being the largest (2.22 cm).

**Table 1:** Characteristics of patients and lesions.

Characteristic			All lesions (n=398)		Lesions≤2 cm (n=250)		Lesions>2 cm (n=148)
**Patient age (years)**							
	Mean (SD)	43.10 (12.57)		43.62 (11.875)		42.22 (13.66)	
	Median (range)	45 (17-70)		45.0 (17-70)		44.5 (17-70)	
**Size of all lesions (cm)**							
	Mean (SD)	1.92 (1.26)		1.1629 (0.42)		3.1876 (1.19)	
	Median (range)	1.6 (0.26-9.5)		1.1 (0.26-2.0)		2.8 (2.1-9.5)	
**Size of malignant lesions (cm)**							
	Mean (SD)	2.22 (1.08)		1.331 (0.42)		3.02 (0.82)	
	Median (range)	2.11 (0.26-6.2)		1.30 (0.26-2.0)		2.8 (2.1-6.2)	
**Size of benign lesions (cm)**							
	Mean (SD)	1.73 (1.33)		1.10 (0.40)		3.38 (1.50)	
	Median (range)	1.3 (0.4-9.5)		1.0 (0.4-2.0)		2.9 (2.1-9.5)	
**Histologic type of malignant lesions, n (%)**							
	Total	150 (37.7)		71 (47.3)		79 (52.7)	
	Intraductal carcinoma in situ	5 (3.3)		5 (7.0)		0 (0.0)	
	Invasive lobular carcinoma	11 (7.3)		10 (14.1)		1 (1.3)	
	Mucinous adenocarcinoma	2 (1.3)		2 (2.8)		0 (0.0)	
	Medullary carcinoma	2 (1.3)		1 (1.4)		1 (1.3)	
	Invasive ductal carcinoma	130 (86.7)		53 (74.6)		77 (97.5)	
**Histological type of benign lesions, n (%)**							
	Total	248 (62.3)		179 (72.2)		69 (27.8)	
	Intraductal papilloma	29 (11.7)		29 (16.2)		0 (0.0)	
	Granulomatous mastitis	5 (2.0)		1 (0.6)		4 (5.8)	
	Fibroma	171 (69.0)		110 (61.5)		61 (88.4)	
	Hyperplasia-induced lesions	42 (16.9)		38 (21.2)		4 (5.8)	
	Scar tissue	1 (0.4)		1 (0.6)		0 (0.0)	

### Reader Performance

**Figure 1:**
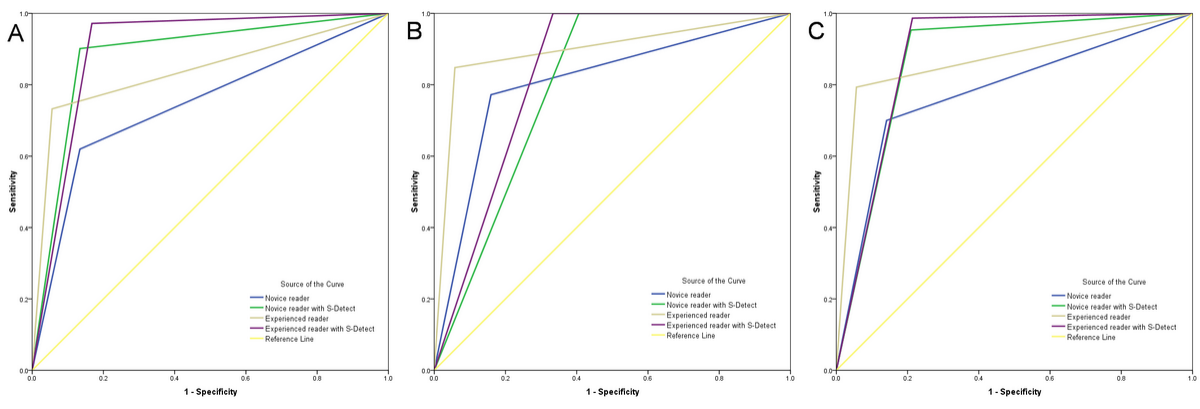
Comparison of receiver operating characteristic curves in lesions <2 cm, lesions >2 cm, and lesions in all size.

In all lesions, the AUCs of the reading improved at combined-CAD mode compared to those of the without-CAD mode for both the novice and experienced reader (Table 2, Figure 1). For the novice reader, the improvement in AUCs was significant between the without-CAD and combined-CAD modes (*Z*=4.90, *P*<.001), whereas there was no significant difference in AUCs between modes for the experienced reader (*Z*=1.06, *P*=.29).

In subgroup analysis, for lesions≤2 cm, the AUCs of the reading improved significantly in combined-CAD mode for both the novice and experienced readers. However, in lesions>2 cm, there were no significant differences in AUCs between two reading modes for both the novice and experienced readers (Table 2).

When a BI-RADS category 4A threshold was used, the sensitivity and NPV improved at the combined-CAD mode compared with that at the without-CAD mode for both the novice reader and experienced reader in all lesions and subgroup analyses (Table 2). However, in lesions≤2 cm, there were no significant differences between without-CAD and combined-CAD modes for the novice reader with respect to specificity, PPV, and accuracy. By contrast, significant differences were observed for the experienced reader in specificity and PPV, whereas there was no significant difference in accuracy. In lesions>2 cm, there was a significant decrease in specificity and a significant increase in NPV between without-CAD and combined-CAD modes for both readers, and there was a significant decrease in PPV for only the experienced reader. There was a moderate reduction in accuracy between the without-CAD and combined-CAD modes for both readers, and in PPV for the novice reader (Table 2).

**Table 2:** Diagnostic performance of the readers in two reading modes with a Breast Imaging Reporting and Data System Category 4A threshold.

Lesions			Novice Reader												Expert Reader													
			Without CAD^a^					Combined with CAD					*P* value		Without CAD						Combined with CAD						*P* value	
			+		–			+	–						+		–				+		–					
**Lesions≤2 cm**																												
	**Pathology**																											
		+	44		22			64		23					52		9				69		33					
		–	27		157			7		156					19		170				2		146					
	Sensitivity^b^		61.97					90.14					<.001		73.24						97.18						<.001	
	Specificity^b^		87.71					87.15					.83		94.97						81.56						.004	
	PPV^c^		66.66					73.56					.22		85.25						67.65						.005	
	NPV^d^		85.33					95.71					.008		89.95						98.65						.005	
	Accuracy		80.40					88.00					.12		88.8						86						.52	
	AUC^e^ (95% CI)		0.74 (0.67-0.82)					0.88 (0.83-0.93)					<.001		0.84 (0.77-0.90)						0.90 (0.86-0.94)						.002	
**Lesions>2 cm**																												
	**Pathology**																											
		+	61			11		79			28				67			4		79				23				
		–	18			58		0			41				12			65		0				46				
	Sensitivity		77.22					100					<.001		86.67						100						<.001	
	Specificity		84.06					59.42					<.001		96.72						66.66						<.001	
	PPV		84.72					73.83					.05		96.30						77.45						<.001	
	NPV		76.32					100					<.001		88.06						100						<.001	
	Accuracy		80.41					81.08					.86		91.74						84.46						.13	
	AUC (95% CI)		0.81 (0.73-0.88)					0.80 (0.72-0.87)					.81		0.90 (0.84-0.95)						0.83 (0.76-0.91)						.03	
**All lesions**																												
	**Pathology**																											
		+	105			33		143			51				119			13		148				52				
		–	45			215		7			197				31			235		2				96				
	Sensitivity		70					95.33					<.001		79.33						98.66						<.001	
	Specificity		86.69					79.43					.13		94.75						79.03						.001	
	PPV		76.08					73.71					.74		90.15						74.00						.003	
	NPV		82.69					96.57					.001		88.34						98.98						.002	
	Accuracy		80.40					85.43					.35		88.94						86.43						.52	
	AUC (95% CI)		0.78 (0.73-0.83)					0.87 (0.84-0.91)					<.001		0.87 (0.83-0.91)						0.89 (0.85-0.92)						.29	

^a^CAD: computer-aided diagnosis.

^b^Breast Imaging Reporting and Data System assessment categories 4B, 4C, and 5 were considered positive for cancer for the calculation of sensitivity and specificity.

^c^PPV: positive predictive value.

^d^NPV: negative predictive value.

^e^AUC: area under the receiver operating characteristic curve.

### Management of Diagnostic Feature Decision Change

At the combined-CAD mode, the management of diagnostic feature decision changes compared with the without-CAD mode was 164/398 (41.2%) of all ultrasound images for the two readers. For the novice reader, with CAD support, 38/150 (25.3%) of the malignant lesions (20 lesions≤2 cm, 18 lesions>2 cm) were correctly upgraded from category 4A to 4B, whereas none (0%) of the malignant lesions was incorrectly changed from 4B to 4A. In addition, 18/248 (7.3%) benign lesions (12 lesions≤2 cm, 6 lesions>2 cm) were correctly downgraded from category 4B to 4A; however, 36/248 (14.5%) benign lesions (11 lesions≤2 cm, 24 lesions>2 cm) were incorrectly changed from category 4A to 4B.

For the experienced reader, 30/150 (20.0%) malignant lesions (18 lesions≤2 cm, 12 lesions>2 cm) were correctly changed from category 4A to 4B, and only 1 of 79 (1%) malignant lesions (≤2 cm) was incorrectly changed from category 4B to 4A. In addition, 4/248 (1.6%) benign lesions (3 lesions≤2 cm, 1 lesion>2 cm) were correctly downgraded from category 4B to 4A, whereas 38/248 (15.3%) benign lesions (3 lesions≤2 cm, 20 lesions>2 cm) were incorrectly changed from category 4A to 4B.

## Discussion

### Principal Findings

CAD systems have been recently applied to improve diagnostic performance in breast ultrasonography. S-Detect is a CAD system based on a neural network learning algorithm [7], which applies a novel feature extraction technique and vector machine classifier that categorizes breast masses into benign or malignant depending on the suggested feature based on the BI-RADS lexicon [15]. Choi et al [10] recently reported that both experienced and inexperienced readers had significantly higher specificity and AUCs in reading ultrasounds in combination with S-Detect, and the inexperienced reader also showed significant improvement in sensitivity. However, the diagnosis of breast lesions of different sizes is one of the most difficult challenges in clinical practice [16,17]. Radiologists with different levels of experience typically perform breast ultrasound, and thus the usefulness of S-Detect may be different according to experience. For example, radiologists with less experience may have a greater benefit in using S-Detect for the diagnosis of small breast lesions.

In our study, when combining ultrasound reading with S-Detect, both the experienced and novice readers showed significantly higher sensitivity and NPV compared to those obtained without S-Detect, which is in line with the findings of the previous studies for CAD systems mentioned above. In addition, 38/150 (25.3%) and 30/150 (20.0%) breast cancers initially assessed as category 4A by the novice and experienced readers were categorized as probably malignant by S-Detect, regardless of size. Combining the results of S-Detect led to significant improvements in AUCs for both readers in lesions<2 cm. However, in lesions>2 cm, the combination of S-Detect did not confer improvements in accuracy and AUC for either reader.

Our results suggest that S-Detect could be used as an additional tool with breast ultrasound regardless of the experience of the reader, and may help to reduce the misdiagnosis ratio of early-stage breast cancer. Although the sensitivity, NPV, and AUCs were improved, there was no significant improvement in the accuracy of the readers when using S-Detect compared to that obtained by the ultrasound reading alone. This may be due to the fact that both readers already showed high AUC values with ultrasound alone, and therefore there was minimal room for improvement.

### Strengths and Prospects

Our results showed that readers with less experience may benefit more by using S-Detect in detection of smaller breast lesions. Several studies have reported the application of different types of CAD to breast ultrasound [6,18,19]. Overall, these studies showed that the CAD systems promoted the diagnostic performance of breast ultrasound, especially specificity and accuracy. Shen et al [18] argued that CAD systems could be helpful in evaluating fuzzy category 4 lesions. Wang et al [19] suggested that combining CAD with ultrasound was more helpful for inexperienced radiologists than for experienced radiologists owing to greater improvement in the diagnostic performances observed in the inexperienced group. In our study, the sensitivity, NPV, and AUCs of both readers were improved, supporting the idea that S-Detect can reliably provide a second view that can be referred to by readers. High sensitivity is a remarkable superiority of S-Detect, and similar results were reported in some previous studies [20,21]. Compared to these previous studies, there was a relatively smaller proportion of benign lesions in our study and the mean size of lesions in our study was larger. In addition, all patients had a breast mass as the prominent symptom, which may explain the different results. Moreover, since S-Detect provides the final assessment in a dichotomized form of possibly benign and possibly malignant, this factor may have also affected the accuracy of readers in the combined-CAD mode.

**Figure 2:**
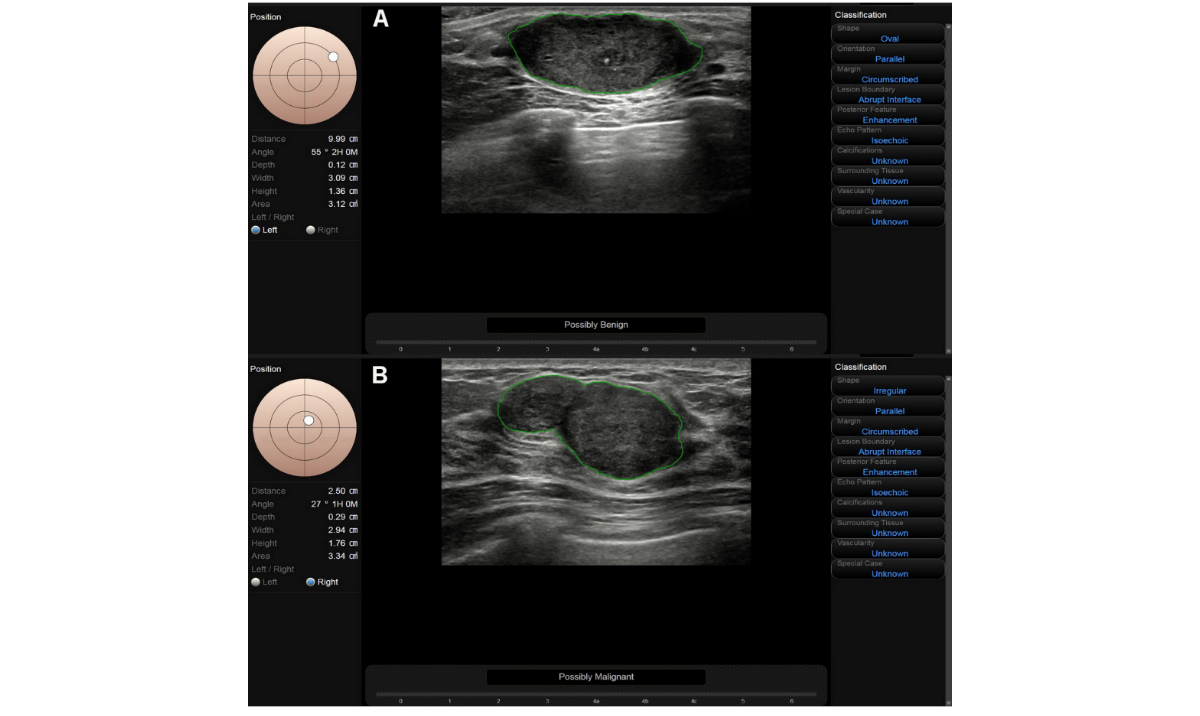
A malignant and benign lesion > 2 cm misdiagnosed by S-Detect.

This result is encouraging for clinical breast cancer screening, as breast cancer is a highly aggressive disease with multiple pathological subtypes, including those associated with higher rates of metastases and poorer survival rates [22]. Thus, it is important to detect cancer early to reduce the mortality rate [23]. In addition, S-Detect is a user-friendly and concise program that is integrated in an ultrasound machine to enable obtaining a terse result for radiologists immediately during real-time ultrasonography, which can easily be applied to routine work. However, it is not recommended to apply CAD alone or use it as a replacement for a radiologist in the diagnosis of breast lesions, especially for tumors>2 cm, which is consistent with the results of Kim et al [13]. As one example from this study, a fibroadenoma lesion with a size of 2.94×1.76 cm (Figure 2A) that showed an unclear margin and a large lobulated shape was misdiagnosed as malignant by S-Detect, and was inversely excluded by the radiologist after combining the results with information on the patient’s history. In another example, a lesion of invasive ductal carcinoma with a size of 3.09×1.36 cm (Figure 2B) showing a clear border and microcalcification was classified as BI-RADS category 4B by conventional ultrasound, whereas S-Detect diagnosed this lesion as benign. Further investigation along with technical progress are anticipated to lead to the development of a more sophisticated algorithm using the multiple-planes assessment BI-RADS ultrasonographic categories.

**Figure 3:**
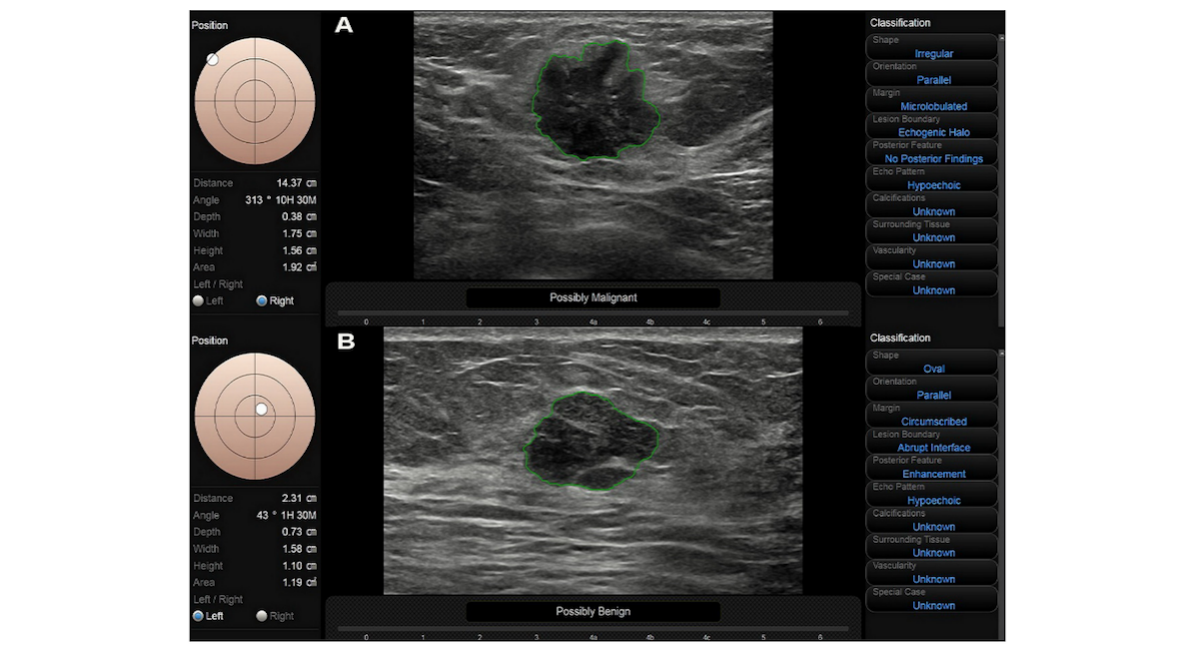
A malignant and benign lesion < 2 cm diagnosed correctly by S-Detect.

Likewise, ultrasound scanning is a real-time and multi-angled imaging method, which can observe the lesion from different planes to collect the imaging features such as the internal situation, relation of the lesion with surrounding tissues, and the blood supply model, along with patient history and other available information. Therefore, more image data and clinical information can be obtained with ultrasound than with CAD. Consequently, in lesions≤2 cm, the combination of S-Detect and ultrasound allows for the weaknesses of each method to be counteracted by the strengths of the other, which could assist both novice and experienced readers in making a more accurate final diagnosis. As one example from this study, an invasive ductal carcinoma lesion with a size of 1.75×1.56 cm (Figure 3A) that showed an unclear margin, irregular shape, and microcalcification was correctly diagnosed as malignant by S-Detect and was classified as BI-RADS category 4C by both readers. In another example, a lesion of fibroadenoma with a size of 1.58×1.10 cm (Figure 3B) showing a clear border and regular shape was classified as BI-RADS category 3 by conventional ultrasound and was correctly diagnosed as benign by S-Detect.

### Limitations

There are several limitations of this study. First, a relatively small number of cases were included (N=261). Second, the presentation of calcifications was not included in the analysis, owing to the limited ability of S-Detect to detect microcalcifications [24]. Third, some small nodules (around 1 cm) classified as BI-RADS category 3 that were not surgically removed were excluded from the study, which may have affected the results. Fourth, only two representative still images of breast masses stored for analysis were chosen during image analysis by the radiologists and S-Detect, which may have caused variability in selecting images of representative planes. Fifth, the criterion of the size of lesions for comparison was set to 2 cm; thus, further studies using other stratifications with a larger number of samples may be warranted. Sixth, both readers had relatively minimal experience as breast imagers. In China, the specialty of breast imaging is somewhat new, and the staff in this field tend to be younger compared with staff of other imaging specialties. Therefore, these factors may have had a slight influence on our results.

### Conclusion

In conclusion, S-Detect is a clinically feasible diagnostic tool that can improve the sensitivity of breast ultrasonography, in addition to improving the NPV and AUC for lesions≤2 cm, with important application value in the clinical diagnosis of breast cancer.

## Abbreviations

AUC: area under the receiving operating characteristic curve
BI-RADS: Breast Imaging Reporting and Data System
CAD: computer-assisted diagnosis
NPV: negative predictive value
PPV: positive predictive value

## References

[1] Siegel RL, Miller KD, Jemal A (2017). Cancer Statistics, 2017. CA Cancer J Clin.

[2] Chen W, Zheng R, Baade PD, Zhang S, Zeng H, Bray F, Jemal A, Yu XQ, He J (2016). Cancer statistics in China, 2015. CA Cancer J Clin.

[3] Brinkley D, Haybrittle JL (1975). The curability of breast cancer. Lancet.

[4] Takahashi R, Kajikawa Y (2017). Computer-aided diagnosis: A survey with bibliometric analysis. Int J Med Inform.

[5] Cho E, Kim E, Song MK, Yoon JH (2018). Application of Computer-Aided Diagnosis on Breast Ultrasonography: Evaluation of Diagnostic Performances and Agreement of Radiologists According to Different Levels of Experience. J Ultrasound Med.

[6] Kim K, Song MK, Kim E, Yoon JH (2017). Clinical application of S-Detect to breast masses on ultrasonography: a study evaluating the diagnostic performance and agreement with a dedicated breast radiologist. Ultrasonography.

[7] Komeda Y, Handa H, Watanabe T, Nomura T, Kitahashi M, Sakurai T, Okamoto A, Minami T, Kono M, Arizumi T, Takenaka M, Hagiwara S, Matsui S, Nishida N, Kashida H, Kudo M (2017). Computer-Aided Diagnosis Based on Convolutional Neural Network System for Colorectal Polyp Classification: Preliminary Experience. Oncology.

[8] Di Segni M, de Soccio V, Cantisani V, Bonito G, Rubini A, Di Segni G, Lamorte S, Magri V, De Vito C, Migliara G, Bartolotta TV, Metere A, Giacomelli L, de Felice C, D'Ambrosio F (2018). Automated classification of focal breast lesions according to S-detect: validation and role as a clinical and teaching tool. J Ultrasound.

[9] Takahashi R, Kajikawa Y (2017). Computer-aided diagnosis: A survey with bibliometric analysis. Int J Med Inform.

[10] Choi J, Kang BJ, Baek JE, Lee HS, Kim SH (2018). Application of computer-aided diagnosis in breast ultrasound interpretation: improvements in diagnostic performance according to reader experience. Ultrasonography.

[11] Liu D, Ba Z, Ni X, Wang L, Yu D, Ma X (2018). Apparent Diffusion Coefficient to Subdivide Breast Imaging Reporting and Data System Magnetic Resonance Imaging (BI-RADS-MRI) Category 4 Lesions. Med Sci Monit.

[12] Spinelli Varella MA, Teixeira da Cruz J, Rauber A, Varella IS, Fleck JF, Moreira LF (2018). Role of BI-RADS Ultrasound Subcategories 4A to 4C in Predicting Breast Cancer. Clin Breast Cancer.

[13] Wang M, Yang Z, Liu C, Yan J, Zhang W, Sun J, Cui G (2017). Differential Diagnosis of Breast Category 3 and 4 Nodules Through BI-RADS Classification in Conjunction with Shear Wave Elastography. Ultrasound Med Biol.

[14] Ma H, Bandos AI, Gur D (2015). On the use of partial area under the ROC curve for comparison of two diagnostic tests. Biom J.

[15] Payne S, Large S, Jarrett N, Turner P (2000). Written information given to patients and families by palliative care units: a national survey. Lancet.

[16] Migowski A (2015). [Early detection of breast cancer and the interpretation of results of survival studies]. Cien Saude Colet.

[17] Guo R, Lu G, Qin B, Fei B (2018). Ultrasound Imaging Technologies for Breast Cancer Detection and Management: A Review. Ultrasound Med Biol.

[18] Shen W, Chang R, Moon WK (2007). Computer aided classification system for breast ultrasound based on Breast Imaging Reporting and Data System (BI-RADS). Ultrasound Med Biol.

[19] Wang Y, Jiang S, Wang H, Guo YH, Liu B, Hou Y, Cheng H, Tian J (2010). CAD algorithms for solid breast masses discrimination: evaluation of the accuracy and interobserver variability. Ultrasound Med Biol.

[20] Morra L, Sacchetto D, Durando M, Agliozzo S, Carbonaro LA, Delsanto S, Pesce B, Persano D, Mariscotti G, Marra V, Fonio P, Bert A (2015). Breast Cancer: Computer-aided Detection with Digital Breast Tomosynthesis. Radiology.

[21] Chabi M, Borget I, Ardiles R, Aboud G, Boussouar S, Vilar V, Dromain C, Balleyguier C (2012). Evaluation of the accuracy of a computer-aided diagnosis (CAD) system in breast ultrasound according to the radiologist's experience. Acad Radiol.

[22] Harbeck N, Gnant M (2017). Breast cancer. Lancet.

[23] Wang Y, Fan W, Zhao S, Zhang K, Zhang L, Zhang P, Ma R (2016). Qualitative, quantitative and combination score systems in differential diagnosis of breast lesions by contrast-enhanced ultrasound. Eur J Radiol.

[24] Choi EJ, Choi H, Park EH, Song JS, Youk JH (2018). Evaluation of an automated breast volume scanner according to the fifth edition of BI-RADS for breast ultrasound compared with hand-held ultrasound. Eur J Radiol.

